# By-Product Formation in Repetitive PCR Amplification of DNA Libraries during SELEX

**DOI:** 10.1371/journal.pone.0114693

**Published:** 2014-12-09

**Authors:** Fabian Tolle, Julian Wilke, Jesper Wengel, Günter Mayer

**Affiliations:** 1 LIMES Institute, University of Bonn, Bonn, Germany; 2 Nucleic Acid Center, Department of Physics, Chemistry and Pharmacy, University of Southern Denmark, Odense M, Denmark; Imperial College London, United Kingdom

## Abstract

The selection of nucleic acid aptamers is an increasingly important approach to generate specific ligands binding to virtually any molecule of choice. However, selection-inherent amplification procedures are prone to artificial by-product formation that prohibits the enrichment of target-recognizing aptamers. Little is known about the formation of such by-products when employing nucleic acid libraries as templates. We report on the formation of two different forms of by-products, named ladder- and non-ladder-type observed during repetitive amplification in the course of *in vitro* selection experiments. Based on sequence information and the amplification behaviour of defined enriched nucleic acid molecules we suppose a molecular mechanism through which these amplification by-products are built. Better understanding of these mechanisms might help to find solutions minimizing by-product formation and improving the success rate of aptamer selection.

## Introduction

Aptamers are short single stranded oligonucleotides with the ability to bind their target with high affinity and high specificity. They are selected against a broad range of targets including small molecules and proteins [1]–[3]. Therefore, aptamers can be conducive to a myriad of applications in various fields such as pharmaceutical sciences, e.g. as therapeutics, for drug discovery, as delivery and targeting units [4], [5], diagnostics, e.g. as sensors [6]–[9] or as tools in chemical biology [10]–[12].

Aptamers are usually generated by an iterative *in vitro* selection process called “systematic evolution of ligands by exponential enrichment” (SELEX)[1], [3], [13]. In a typical SELEX experiment a library of up to 10^15^ different nucleic acid strands is exposed to a selection pressure were the best target binding sequences are partitioned from the rest. The best binding sequences are recovered, amplified by PCR and the enriched library is employed in the next selection cycle. After several rounds of selection and amplification the library is sequenced and monoclonal aptamer sequences are identified.

SELEX libraries typically comprise up to 100 nucleotides in length, containing a random region flanked by two constant primer-binding sites, which are used for PCR amplification of the library. The presence of up to 10^15^ different strands with undefined random regions can lead to several issues such as by-product formation in the PCR amplification. In conventional PCR approaches the major source for by-product formation is primer-primer hybridization leading to shorter double stranded products. In contrast to that, during amplification of complex libraries, single stranded sequences can bind to complementary bases in the random region serving as a primer, which is then extended by the polymerase, yielding longer ss-dsDNA products [4], [14], [15]. This mechanism can lead to a full conversion of the product to longer by-products, even before the primers are exhausted.

Although being a common problem in the amplification of complex libraries [6], [8], [9], [16], [17], especially in the *in vitro* evolution community only a few studies about the molecular constitution and the underlying mechanisms of by-product formation have been published. In one example Marshall and Ellington reported molecular parasites enriched during a continuous isothermal amplification reaction that are longer than their parental molecules, yet replicate much more efficiently [10]. To our knowledge, albeit being detrimental to the enrichment process, there is no published report yet describing the mechanism for the formation of longer by-products during PCR amplification in SELEX experiments.

Herein we report two special kinds of PCR by-products observed during SELEX experiments conducted in our laboratory. Based on sequencing data we suggest a mechanistic model for the generation and amplification of this kind of by-products.

## Material and Methods

### Chemicals and Materials

All chemicals and the single-strand binding protein were purchased from Sigma-Aldrich (Munich, Germany). Human activated protein C was purchased from CellSystems (Troisdorf, Germany). Streptavidin coupled magnetic beads (Dynabeads M280) were purchased from Life Technologies (Darmstadt, Germany). Lambda exonuclease and T4 polynucleotide kinase were purchased from New England Biolabs (Frankfurt, Germany). Gamma ^32^P ATP was purchased from PerkinElmer (Rodgau, Germany). NHS-PEG4-Biotin was purchased from Thermo Scientific (Rockford, USA). DNA purifications were done with NucleoSpin Clean-Up/Plasmid kits from Macherey-Nagel (Düren, Germany) according to the manufacturers recommendation.

### Oligonucleotides

Library: 5′-TGACCTTCG**T**G**A**C**T**ACAGNNNNNNNNNNNNNNNNNNNNACTTAGTCAACT-GGCACAAT-3′; Forward primer: 5′-TGACCTTCG**T**G**A**C**T**ACAG-3′; Reverse primer: 5′-Phosphate-ATTGTGCCAGTTGACTAAGT-3′; Bold bases represent LNA building blocks. For subsequent lambda exonuclease digestion the reverse primer is labelled with a 5′ phosphate group. Prior each selection cycle or binding assay the DNA was heat conditioned (refolded) by heating to 95°C for 3 min and slowly cooling down to 25°C in D-PBS.

### PCR

PCR was done in a Mastercycler Personal/Gradient from Eppendorf (Hamburg, Germany). GoTaq DNA polymerase from Promega (Mannheim, Germany) and the supplied buffer containing 5 mM Mg^2+^ was use with the following cycling program (5 min 95°C; 1 min 95°C, 1 min 54°C, 1 min 72°C; hold 4°C). The optimal Mg^2+^ concentration and annealing temperature for the library were screened prior selection. The Mg^2+^ concentration was screened from 1.5 mM–5 mM, with increasing concentrations yielding more PCR-product and the highest product yield at 5 mM Mg^2+^ (S1 Figure). The annealing temperature was screened from 45°C to 65°C. 54°C was chosen as the optimal annealing temperature being the highest temperature to give optimal yields due to strongly decreasing yields from 57°C upwards (S1 Figure). 0.5 µM of both primers and 250 µM of dNTPs were used.

### SELEX

The selections were carried out in selection buffer based on Dulbeccos' phosphate buffered saline (D-PBS) enriched with 1 mg/ml bovine serum albumin (BSA).

As target activated protein C (APC) immobilized on magnetic beads was used. For the selection, 100 µg APC were biotinylated with NHS-PEG4-Biotin and immobilized on 5 mg streptavidin coated magnetic beads. 500 pmol of the start library were incubated with 500 µg of empty streptavidin beads to remove the unwanted matrix binding sequences. The supernatant was incubated for 30 min at 37°C with 500 µg APC loaded beads in a total volume of 200 µl. The beads were thoroughly washed two times (30 sec and 5 min) with 200 µl selection buffer to remove unspecific binding DNA and the enriched library was eluted with 85 µl water from the beads by heat denaturation (80°C for 3 min). The eluted DNA was PCR amplified and purified via spin-columns. PCR product formation (18–22 PCR cycles) was carefully monitored by gel electrophoresis and stopped as soon as the desired product band could be detected on ethidium bromide stained gels. Single strand displacement of the double stranded PCR product was carried out by lambda exonuclease digestion with 25 U lambda exonuclease [16], [17] for 1 h at 37°C and the resulting single stand DNA was purified via spin-columns. The purified single stand DNA (approx. 10 pmol) was applied in the next SELEX round. For each selection cycle the PCR product and the purified single strand DNA were analysed by gel electrophoresis on a 4% agarose gel. To increase the selection pressure the stringency of the washing steps was gradually increased by 2 to a maximum of 8 washing steps during the selection.

### Cloning and Sequencing

Cloning was done with a TOPO TA Cloning kit from Life Technologies (Darmstadt, Germany) according to the manufacturers recommendation. 24 clones were sent to GATC-Biotech (Konstanz, Germany) for Sanger sequencing.

### Binding Affinity Assay

Equilibrium dissociation constants were determined by nitrocellulose filter retention with radioactive labelled DNA. The single stranded DNA was 5′ labelled with ^32^P from gamma ^32^P ATP using T4 polynucleotide kinase. The labelled DNA was purified from unreacted ATP and incubated in selection buffer with increasing concentrations of APC for 30 min at 37°C. The solution was filtered through nitrocellulose and the amount of bound aptamer was quantified by autoradiographie in a phosphorimager (FLA 5100 from Fuji, Japan). Raw binding data were corrected for unspecific background binding and equilibrium dissociation constants were calculated via nonlinear regression assuming a 1∶1 binding model with Prism 5.0f (GraphPad Software, La Jolla, USA).

## Results and Discussion

This study originated in the attempt to select DNA aptamers from constrained libraries with pre-defined stem regions. By pre-defining stem structures in the primer binding sites we endeavour to select aptamers that can easily be truncated in a post-SELEX maturation step, resulting in a fast and reliable method for the generation of short and versatile employable aptamers. Encouraged by the successful results previously obtained with RNA libraries [13] we set out to select compact DNA aptamers with a very short, only 6 bp long stem region, which is stabilized by three “Locked Nucleic Acids” (LNA) [14], [15] building blocks. As a model target the serine protease activated protein C (APC), which plays an important role in blood clotting, inflammation and cell death, was chosen.

**Figure 1:**
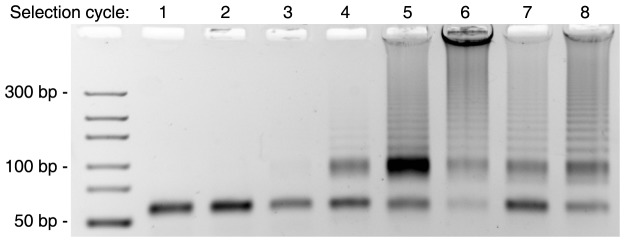
Agarose gel analysis of the PCR products of the 8 SELEX cycles (lanes 1–8) showing the ladder-type and non-ladder-type by-products. Visualization by ethidium bromide staining; 4% agarose gel.

During the course of a SELEX experiment we observed an emerging by-product pattern. From the 4^th^ round on, two different kinds of dsDNA by-products after PCR amplification were observed (Fig. 1). Besides the correctly sized product band reflecting the length of the 58 base pair (bp) comprising dsDNA staring library, an intense second band migrating between the 75 bp and 100 bp marker lanes was observed. Additionally, a ladder-like product pattern was observed. Despite this by-product formation the SELEX was carried on for a total of eight rounds. After the 5^th^ round a gel extraction of the correctly sized (58 bp) product band was performed in order to eliminate the by-products. However, despite this effort the same by-product pattern emerged after the next PCR amplification round. Subsequent filter retention binding assays with the 8^th^ round library showed, as expected, no enrichment of target binders. This finding demonstrates that by-product formation is detrimental to a successful enrichment process.

In order to rule out that the nature of the library, containing LNAs caused this pattern formation, a control experiment with unmodified DNA primers was performed (S2 Figure). After consecutive amplification of the unmodified DNA library the same by-product pattern could be observed, showing that by-product formation is not due to the use of LNA-containing primer binding sites.

Similar by-product patterning has been previously observed in our laboratory using various nucleic acid libraries. In one example a selection against a small organic molecule with a different library containing a pre-defined stem (5′-AGCATAGAGACATCTGCTATTGGTAGCACA-N25-TGTGCAACCGTAGACTCCAGACTTCAGGTA-3′) was rendered unsuccessfully due to the formation of similar ladder type by-products. Albeit vigorous gel purification attempts no enrichment could be observed. In our experience by-product formation is especially pronounced in constrained libraries, but not restricted to this kind of libraries only. The same by-product pattern was also observed with two other conventional unstructured libraries (5′-GCCTGTTGTGAGCCTCCTAAC-N49-CATGCTTATTCTTGTCTCCC-3′ and 5′-CACGACGCAAGGGACCACAGG-N42-CAGCACGACACCGCAGAGGCA-3′) (S3 Figure). This is especially noteworthy since both libraries were previously successfully used in other selection experiments [2], [18].

**Figure 2:**
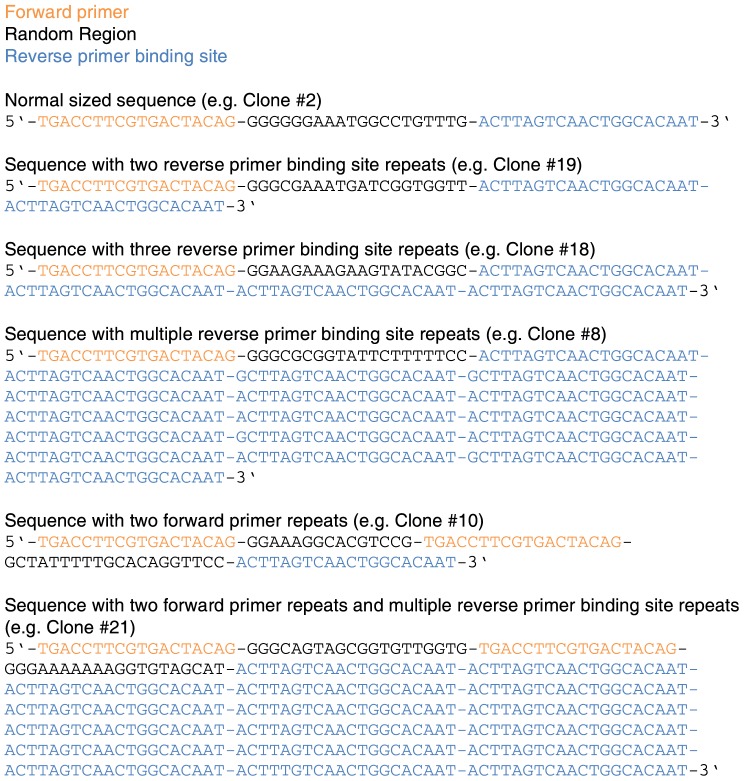
Cloning and sequencing of the library from selection cycle 8 yielded different classes of oligonucleotide sequences.

Having observed ladder type by-products in several selection attempts, we set out to investigate the nature of this kind of by-products. To understand why such elongated by-products are preferentially formed, the 8^th^ round SELEX library was cloned and 24 clones were sent for sequencing. Representative sequences of the different classes of by-products identified are depicted in Fig. 2 (for a complete list of sequencing data please refer to the S4 Figure).

Sequencing revealed two types of by-product sequences. One set of sequences showing two copies of the forward primer linked by stretches of random region. The length and the sequence of the random stretch vary between the sequences. Another set of sequences shows a direct fusion of multiple full-length reverse primer binding sites fused without gap. With the sequence information in hand we were able to propose a mechanism that explains the formation of both types of by-products.

**Figure 3:**
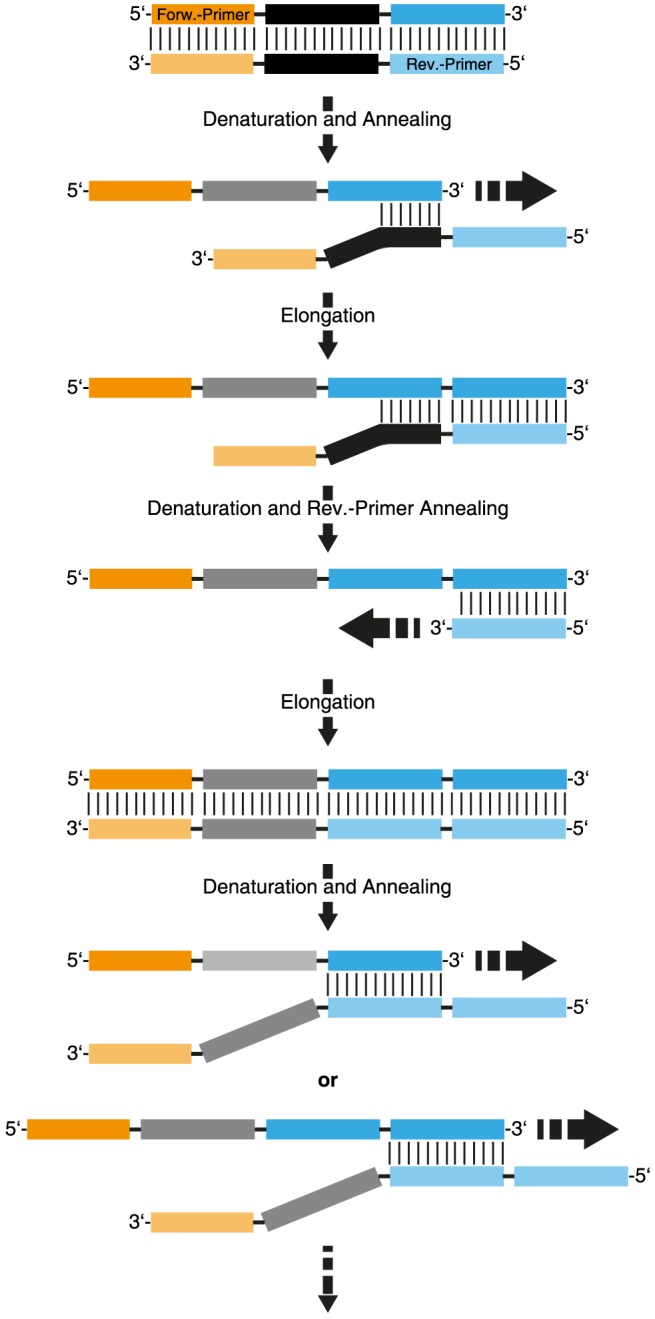
Proposed mechanism of “ladder type” by-product formation.

**Figure 4:**
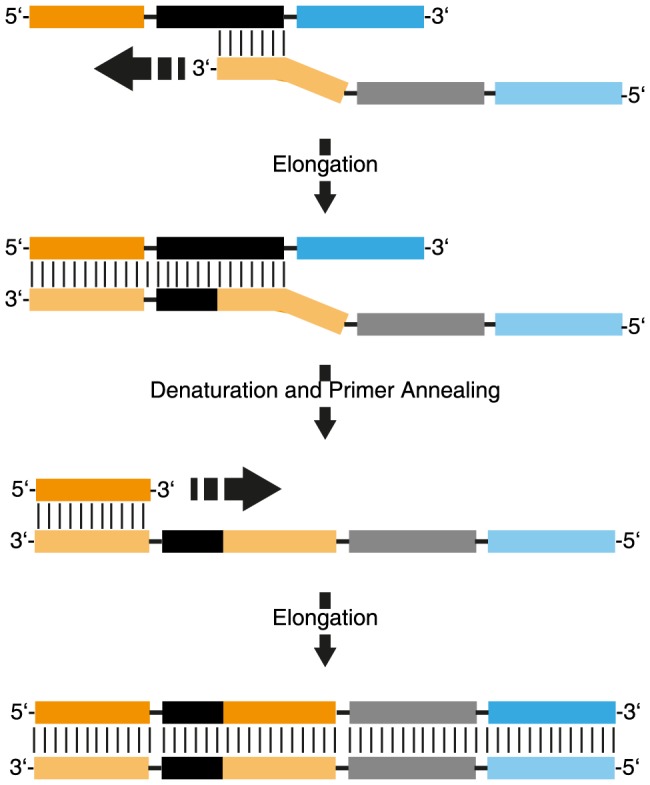
Proposed mechanism of “non-ladder type” by-product formation.

In this model, annealing of the 3′ end of a random sequence to a complementary part in the random region on another strand acts as a primer and leads to the elongation of this strand. Amplification of this strand in the next PCR cycle can lead to a double stranded DNA product with doubled reverse primer binding sites (Fig. 3). This by-product can be amplified by PCR and subsequently acts as a template for the formation of strands with multiple copies of reverse primer binding sites. Since long stretches of such repeats allow for an efficient annealing of DNA, ever-longer by-products can be formed and will eventually dominate the library composition (Fig. 3). This mechanism leads to increasingly longer by-products but is only possible when a gap free full extension of the primer-binding site occurs. If the initial priming event happens in the middle of the random region (Fig. 4), the resulting by-product will have random gaps, preventing it to act as a template for further extensions.

**Figure 5:**
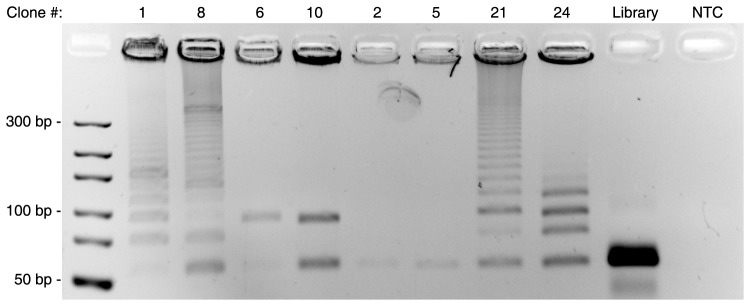
PCR amplification of representative mono-clones, the start library and a non-template control (NTC). Visualization by ethidium bromide staining; 4% agarose gel.

In order to test our proposed by-product formation mechanism we conducted PCR amplification experiments with some of the representative monoclonal sequences (Fig. 5). In agreement with the proposed mechanism, the clones that reflect the length of the initial library (clones 2 and 5) yielded only one product of the expected size. However, amplification of clones with multiple reverse primer binding sites yielded ladder type by-products (clones 1, 8, 21, and 24). In turn, clones with a duplication of the 5′ region (6 and 10), supposedly formed by the mechanisms described in Fig.4, showed two products, but no formation of the ladder type by-products.

Having observed the ladder type by-products in several cases we believe that understanding the mechanism leading to the formation of this type of by-products is of general interest for the SELEX community and that the insights we could obtain might help to find solutions for this problem.

In a first attempt to overcome, or at least reduce, by-product formation several optimization attempts were executed. To disfavour the formation of long PCR products a special PCR program, completely skipping the elongation step, was investigated (5 min 95°C; 1 min 95°C, 30 sec 54°C; hold 4°C). Using this amplification strategy in consecutive PCR reactions the formation of ladder type by-products could be delayed to the 6. cycle.

Furthermore the use of single strand binding protein (SSB) was explored. In a concentration of 15 ng/µl the addition of SSB could delay the formation of ladder type by-products by one cycle, again alleviating, but not solving the problem. Additionally a study employing a shorter, only 13 nt long, reverse primer was employed. This time no improvement could be observed.

Since the addition of SSB and the optimized PCR program showed some improvement in delaying the formation of by-products in consecutive PCR cycles, a new SELEX attempt was started. Unfortunately despite the effort to reduce PCR by-products, also this attempt had to be aborted after 4 cycles of selection due to the formation of ladder type by-products.

**Figure 6:**
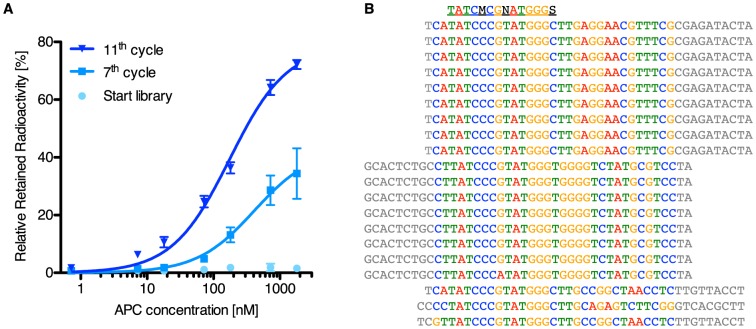
Filter retention assay, A), with radioactive labelled DNA from the start library, 7^th^ and 11^th^ selection cycle. A strong increase in binding affinity is observable, demonstrating a successful enrichment during the SELEX. **B**) Sequencing data from the 11^th^ selection cycle without primer binding sites. All mono-clones share the previously for high affinity APC aptamers described consensus motive (5′-TATCMCGNATGGGS-3′) [11], [12], [19].

To rule out a general problem with our SELEX methodology, a selection with a library containing different primer binding sites was carried out (5′-CACGACGCAAGGGACCACAGG-N42-CAGCACGACACCGCAGAGGCA-3′). In this case strong enrichment was observed (Figure 6A), thereby proving our SELEX methodology itself to be very effective for the selection of aptamers against APC. Cloning and sequencing the library of the elevens selection cycle revealed three families of sequences. Interestingly, all sequences identified share the same consensus motive that was previously described for high affinity aptamers against APC [5], [19] (Figure 6B).

In conclusion, we demonstrate that amplification reactions during SELEX experiments can yield artificial by-product formation, which can be overcome by primer design. Our data reveal that these by-products build according to a special base-pairing mechanism, which results in distinct amplification patterns. Importantly, enrichment of specific target sequences is prohibited, most likely due to loss of potential binding sequences. So far all our attempts to reduce the by-product formation with this library failed, but the selection with a library containing different primer binding sites was successful. Noteworthy, in a different context, repetitive amplification of this library eventually also led to by-product formation (S3 Figure). This is an indication that besides primer design, the target also plays an important role. Since some desired library/target pairs are especially problematic, more solutions to generally increase the likelihood of a successful SELEX in these difficult cases are needed. Maybe the amplification related problems during SELEX might be overcome by special PCR methods and tweaked library design. Especially emulsion PCR has proven to be successful for the reduction of by-product formation in complex mixtures [7], [20]-[22] and should be further investigated in this context.

## Supporting Information

S1 Figure **Optimisation of PCR conditions prior selection.** **A)** The Mg^2+^ concentration was screened from 1.5 mM–5 mM **B)** The annealing temperature was screened from 45°C to 65°C. Visualization by ethidium bromide staining; 4% agarose gel.
(EPS)

S2 Figure **Comparison of by-products using LNA and DNA primer in consecutive rounds of amplification.** In both cases ladder-type by-products can be observed after 4 rounds of consecutive amplification. Visualization by ethidium bromide staining; 4% agarose gel.
(EPS)

S3 Figure **By-product formation observed with another library (5′-CACGACGCAAGGGACCACAGG-N42-CAGCACGACACCGCAGAGGCA-3′) during a different selection experiment.** Visualization by ethidium bromide staining; 4% agarose gel.
(EPS)

S4 Figure **Complete list of sequencing data.** Clones 4, 7, 13 and 20 did not yield utilisable sequencing results.
(EPS)
